# Long-term outcomes of lung transplantation with *ex vivo* lung perfusion technique

**DOI:** 10.3389/frtra.2024.1324851

**Published:** 2024-02-06

**Authors:** Sana N. Buttar, Hans Henrik L. Schultz, Hasse Møller-Sørensen, Michael Perch, Rene Horsleben Petersen, Christian H. Møller

**Affiliations:** ^1^ Department of Cardiothoracic Surgery, Copenhagen University Hospital, Rigshospitalet, Copenhagen, Denmark; ^2^ Department of Clinical Medicine, University of Copenhagen, Copenhagen, Denmark; ^3^ Department of Cardiology, Section for Lung Transplantation, Copenhagen University Hospital, Rigshospitalet, Copenhagen, Denmark; ^4^ Department of Cardiothoracic Anaesthesiology, Copenhagen University Hospital, Rigshospitalet, Copenhagen, Denmark

**Keywords:** *ex vivo* lung perfusion (EVLP), EVLP donor lungs, non-EVLP donor lungs, chronic lung allograft dysfunction, EVLP protocols

## Abstract

*Ex vivo* lung perfusion (EVLP) has demonstrated encouraging short- and medium-term outcomes with limited data available on its long-term outcomes. This study assesses (1) EVLP long-term outcomes and (2) EVLP era-based sub-analysis in addition to secondary outcomes of recipients with EVLP-treated donor lungs compared with recipients of conventionally preserved donor lungs in unmatched and propensity score-matched cohorts. Double lung transplants performed between 1st January 2012 and 31st December 2021 were included. A total of 57 recipients received EVLP-treated lungs compared to 202 unmatched and 57 matched recipients who were subjected to non-EVLP-treated lungs. The EVLP group had a significantly lower mean PaO_2_/FiO_2_ ratio and significantly higher mean BMI than the non-EVLP group in the unmatched and matched cohorts. The proportion of smoking history in the unmatched cohort was significantly higher in the EVLP group, while a similar smoking history was demonstrated in the matched cohorts. No difference was demonstrated in overall freedom from death and retransplantation between the groups in the unmatched and matched cohorts (unmatched: hazard ratio (HR) 1.28, 95% confidence interval (CI) 0.79–2.07, *P* = 0.32; matched: HR 1.06, 95% CI 0.59–1.89). *P* = 0.89). In the unmatched cohort, overall freedom from chronic allograft dysfunction (CLAD) was significantly different between the groups (HR 1.64, 95% CI 1.07–2.52, *P* = 0.02); however, the cumulative CLAD incidence was similar (HR 0.72, 95% CI 0.48–1.1, *P* = 0.13). In the matched cohort, the overall freedom from CLAD (HR 1.69, 95% CI 0.97–2.95, *P* = 0.06) and cumulative CLAD incidence (HR 0.91, 95% CI 0.37–2.215, *P* = 0.83) were similar between the groups. The EVLP era sub-analysis of the unmatched cohort in 2012–2014 had a significantly higher cumulative CLAD incidence in the EVLP group; however, this was not demonstrated in the matched cohort. All secondary outcomes were similar between the groups in the unmatched and matched cohorts. In conclusion, transplantation of marginal donor lungs after EVLP evaluation is non-detrimental compared to conventionally preserved donor lungs in terms of mortality, retransplantation, cumulative CLAD incidence, and secondary outcomes. Although the unmatched EVLP era of 2012–2014 had a significantly higher cumulative CLAD incidence, no such finding was demonstrated in the matched cohort of the same era.

## Introduction

Lung transplantation (LTx) remains a definitive treatment for end-stage pulmonary disease with a demonstration of persistently encouraging early outcomes (1). As a result, the demand for LTx has increased significantly, leading to a critical shortage of suitable donor lungs (2). The mortality rate among individuals on the waiting list for LTx is reported to be in the range of 15%–30%, which has prompted the lung transplant community to explore additional means to expand the donor lung pool (3–5). One of the measures taken into account is the loosening of “standard criteria” for donor lungs with the introduction of “extended criteria” donor lungs to be used for LTx (6, 7). However, despite this initiative, only 20% of the donor lungs are transplanted, with this low transplant rate attributed to the uncertainty of donor lung function and concern about their risk of developing primary graft dysfunction (5, 8).

The *ex vivo* lung perfusion (EVLP) technique has been established as a method to re-evaluate the donor lung function in addition to improving and potentially treating marginal donor lungs, which otherwise would have been discarded (9–12). Accordingly, this enables the ability of addressing the immediate concern of uncertain lung function under the control of a transplant team, with further opportunity of ameliorating and repairing the donor lungs and thereby increasing the much-required donor pool (13). Indeed, EVLP has demonstrated the ability to significantly increase the lung transplant rate in high-volume centers regardless of different EVLP protocols suggesting the invaluable benefits and future potential of the technique (4, 7, 13–18).

The majority of the current evidence demonstrates short-term and medium-term outcomes for recipients of EVLP-treated donor lungs compared with recipients of conventionally preserved donor lungs (4, 15–23). This in contrast to the limited number of studies investigating long-term follow-up outcomes between these two groups (13, 24–26). The primary aim of this single-center study was to ascertain the long-term outcomes, such as overall freedom from death and retransplantation and overall freedom from chronic allograft dysfunction (cumulative CLAD incidence), of recipients with EVLP-treated donor lungs compared with recipients of non-EVLP treated donor lungs. EVLP era-based sub-analysis and secondary outcomes were also assessed between these two groups.

## Materials and methods

Data were collected and analyzed retrospectively. The *Scandia Transplant Program* database was searched for patients who underwent LTx between 1st January 2012 and 31st December 2021 at Copenhagen University Hospital, Rigshospitalet, Denmark. This center has been centralized to perform nationwide conventional LTx since 1992. In May 2012, the EVLP technique was introduced in Denmark with additional nationwide EVLP activity concentration and subsequent LTx in the center.

All donor lungs were procured according to our standard protocol by our retrieval team of surgeons. Before 2020, all donor lungs were flushed with 3 L of antegrade Custodiol® (HTK solution, Bensheim, Germany) without a routine retrograde flush. The protocol was subjected to change in 2020 with an antegrade cold Perfadex flush (XVIVO AB, Gothenburg, Sweden) of 3 L and a routine retrograde cold Perfadex flush of 1 L before and after the lung harvest, respectively. No other change was made to the EVLP protocol during the study period. The lungs were stored in cold saline and kept on ice during transport to our center. Donor lungs were allocated to recipients on the basis of blood group, total lung capacity, and wait-list status (urgency). The decision to evaluate rejected marginal donor lungs on EVLP was made by the transplant team. All lungs were from donors after brain death as donation after circulatory death has only recently been approved by the Danish authorities.

All recipients received the same post-transplant care during the study period, including immunosuppressive treatment in keeping with our usual practice. After discharge, a routine clinical follow-up occurred as follows: weekly during the first 3 months; every month for the first year; every 2 months for the second year; and then quarterly throughout their life. Routine clinical assessment included pulmonary function tests, chest radiography, and blood tests. Routine transplant bronchoscopies with biopsy samples were performed at 2, 4, and 6 weeks followed by 3, 6, 12, 18, and 24 months.

### Donor inclusion and exclusion criteria

All donor lungs fulfilling the standard LTx criteria were transplanted directly (27). Lungs rejected for standard transplantation were evaluated on EVLP if: (1) systemic arterial oxygenation (PO_2_) ≤ 40 kPa on fraction of inspired oxygen (FiO_2_) of 1.0 with positive end-expiratory pressure (PEEP) of 5 mmHg equivalent to a partial pressure of arterial oxygen/fraction of inspired oxygen (P/F) ratio of ≤301 mmHg. If PO_2_ ≥ 40 kPa on FiO_2_ of 1.0, lungs were further tested with PO_2 _≤ 13 kPa on FiO_2_ of 0.4 (P/F ratio of ≤244 mmHg) as a continued indication for EVLP; and (2) severely impaired lungs on anamnestic/radiology/macroscopic assessment. The primary reason for all marginal lungs evaluated on EVLP was inadequate P/F with no contraindication. As per standard protocol, the first P/F ratio sample was taken without the lung recruitment. If the P/F ratio was ≤301 mmHg, an attempt was made to recruit the lungs in atelectatic portions. If the lung recruitment was successful with increase in P/F ratio equivalent to standard LTx criteria, the lungs were excised from the donor and transplanted directly. If not, the lungs were evaluated on EVLP. Lung recruitment was performed by a PEEP of no more than 6 cm H_2_O for 30 seconds (s). Lungs after EVLP were selected for transplantation if the following criteria were met: (1) PO_2_ > 50 kPa on FiO_2_ of 1.0 or >13 kPa on FiO_2_ 0.21 measured directly from right and left lung veins; (2) stable or improving lung compliance; (3) stable or falling pulmonary vascular resistance (PVR); (4) no major pathology on lung inspection and palpation; and (5) a positive collapse test. Donor lungs with severe established pneumonia and evidence of gastric aspiration were excluded.

### Recipient inclusion and exclusion criteria

All patients on our lung transplant waiting list, including patients bridged for transplant with invasive mechanical ventilation and extracorporeal life support (ECMO) were eligible for lung transplantation, with or without EVLP. Patients who underwent combined heart-lung transplantation, single-lung transplantation, and retransplantation with a primary lung transplant before 2012 were excluded. Patients with primary lung transplants conducted after 2011 with retransplantation as an outcome were included.

### EVLP protocol

The EVLP technique used in our center has been described previously (28). In brief, vivoline LS 2 2015 model (Vivoline Medical AB, Lund, Sweden) was used for the perfusion. The system was primed with 2 L of STEEN solution (XVIVO Perfusion AB, Gothenburg, Sweden) and mixed with red blood cells to a hematocrit level of 10%–15%. Lung perfusion flow was limited to 70 mL/min/kg donor weight. The pulmonary artery (PA) pressure limit was gradually increased to a maximum of 20 mmHg during the perfusion. At 32°C, mechanical volume-controlled ventilation was applied with a continuous PEEP level of 5 cm H_2_O and incrementally changing tidal volume of 4–8 mL/kg donor weight according to the temperature and perfusion phase. Repeated blood samples for gas analysis were drawn from the left atrium and compared with simultaneous samples from the PA. The pCO_2_ in the deoxygenated blood did not exceed 6 kPa. Lungs on EVLP were evaluated hourly with a maximum perfusion time of 3 hours (h). Lungs were eligible for transplantation upon first evaluation achieving the post-EVLP criteria. If the post-EVLP criteria were still not fulfilled after the third evaluation, the lungs were discarded. A collapse test was performed by disconnecting the tracheal tube at the end of inspiration. Recoil was subjectively evaluated. Lung compliance and PVR were continuously monitored. Accepted lungs were cooled in the EVLP system before transplantation. The ventilation was stopped at 32°C with a continued PEEP level of 5 cm H_2_O. The perfusate flow through the lungs was exchanged for topical cooling at 15°C preceded by a cold flush with 2 L of Perfadex and a target temperature of 8°C.

The total preservation time for EVLP and non-EVLP lungs was defined as the clamping of pulmonary artery and start of pulmoplegia in the donor to the release of pulmonary artery clamp in the recipient.

### Study end points

Primary outcomes were allograft survival (freedom from death and retransplantation from all causes) and freedom from chronic lung allograft dysfunction (CLAD) (cumulative CLAD incidence). CLAD was defined according to the International Society of Heart and Lung Transplantation criteria as a decline of 20% or more in measured forced expiratory volume in 1 s (FEV_1_) from the post-transplant baseline value (29). The baseline value is defined as the mean of the best two postoperative FEV_1_ measurements taken more than 3 weeks apart (29). Each patient who met the CLAD criteria was screened by two authors (HHS and MP) to validate the diagnosis. The primary outcomes were additionally sub-analyzed based on three different EVLP eras: 2012–2014, 2015–2018, and 2019–2021.

The secondary outcomes included postoperative parameters such as primary graft dysfunction (PGD) > Grade 1 at 72 h, duration of ventilator, intensive care unit (ICU) and hospital stay in addition to incidence of ECMO, tracheostomy, cytomegalovirus (CMV) infection at the third month after transplantation, and biopsy-proven acute rejection episodes if the grade was A3 or higher.

The data were mainly obtained from patient records. The CLAD data were extracted from *Spirotrac 6* software after all other data were collected blinded.

### Statistical analysis

Continuous data are presented as medians with interquartile ranges (IQR) with differences between the groups compared with a Mann‒Whitney *U*-test. Categorical data are presented as frequency with percentage with differences between the groups compared with a Fisher's exact test. Kaplan‒Meier curves were used for overall freedom from death and retransplantation plots and overall freedom from CLAD plots with log-rank test to compare proportional hazards of the plots. An additional competing risk analysis was conducted for CLAD data with a sub-distributed hazard ratio (HR) for the comparison of cumulative CLAD incidence between the groups. Data were analyzed for both the unmatched and matched cohorts of the EVLP and non-EVLP groups. The matched group was created using propensity score matching (PSM), where one recipient of EVLP lungs was paired with one recipient of non-EVLP lungs of best fit based on donor and preoperative recipient characteristics (Tables 1 and 2). The matched group was additionally adjusted for donor smoking and donor body mass index (BMI). A *P* value < 0.05 was considered statistically significant. All statistical analyses were performed using RStudio version 2022.07.2. Matching was performed using the “MatchIt” package (version 4.5.5) in RStudio.

**Table 1:** Donor characteristics.

Variables	Groups				
	Unmatched			Matched	
	EVLP (*n* = 57)	Non-EVLP (*n* = 202)	*P-*value	Non-EVLP (*n* = 57)	*P-*value
Male, no. (%)	35 (61.4)	101 (50)	0.14	31 (54.38)	0.57
Age, median (IQR), year	46 (35–55)	50 (39–57)	0.19	52 (41–58)	0.12
BMI, median (IQR)	26.8 (24.3–29.9)	24.9 (22.5–27.7)	0.006	24.5 (22.4–26.9)	0.008
Smoking history, no. (%)	28 (49.1)	63 (31.2)	0.02	28 (49.1)	1
Chest radiograph abnormality, no (%)	14 (24.6)	42 (20.8)	0.58	7 (12.3)	0.14
Preprocurement PaO_2_:FiO_2_ ratio, median (IQR), mmHg	199 (143–277)	366 (294–435)	<0.001	362 (282.5–363)	0.01
Total preservation time, median (IQR), min^a^	661 (575–766)	390 (300–449)	<0.001	330 (279–416)	<0.001
EVLP time, median (IQR), min	195 (137.3–235)	–	–	–	–
Cause of death, no (%)					
Intracerebral hemorrhage	34 (59.7)	124 (61.4)	0.05	36 (63.2)	0.22
Cerebral infarction	1 (1.7)	11 (5.4)		1 (1.7)	
Brain trauma	2 (3.5)	13 (6.4)		1 (1.7)	
Cerebral anoxia	15 (25.3)	20 (9.9)		2 (3.51)	
Others	5 (9.8)	34 (16.9)		6 (10.5)	

EVLP, *ex vivo* lung perfusion; no., number; %, percent; IQR, interquartile range; y, years; BMI, body mass index.

^a^ Total preservation time is defined as clamping of pulmonary artery and start of pulmoplegia in the donor to the release of pulmonary artery clamp in the recipient.

**Table 2:** Recipient characteristics.

Variables	Groups				
	Unmatched			Matched	
	EVLP (*n* = 57)	Non-EVLP (*n* = 202)	*P-*value	Non-EVLP (*n* = 57)	*P-*value
Male, no. (%)	32 (56.1)	106 (52.4)	0.65	28 (49.1)	0.57
Age, median (IQR), year	55 (44–58)	53 (44–58)	0.49	54 (46–58)	0.76
BMI, median (IQR)	20.8 (18.7–26.2)	21.9 (18.8–26)	0.64	21.1 (18–25.6)	0.89
Diagnosis, no., (%)					
COPD/Emphysema	19 (33.3)	59 (29.2)		22 (38.5)	0.06
Alpha-1-antitrypsin deficiency	3 (5.3)	32 (15.8)		7 (12.2)	
Idiopathic pulmonary fibrosis	10 (17.6)	22 (10.9)		5 (8.8)	
NSIP	4 (7)	13 (6.4)	0.35	1 (1.7)	
Cystic fibrosis	8 (14)	27 (13.4)		9 (15.8)	
Pulmonary arterial hypertension	3 (5.3)	10 (4.9)		1 (1.7)	
Others^a^	10 (17.5)	39 (19.4)		2 (3.5)	
Prioritised listing,^b^ no., (%)	26 (45.6)	91 (45)	0.87	16 (28%)	0.06
Wait-list time, median (IQR), day	144 (55–365)	107 (40.2–281)	0.39	144 (48–345)	0.84
Blood group, no., (%)					
O	20 (35)	81 (40.1)		21 (36.8)	0.37
A	5 (8.8)	30 (14.8)		10 (17.5)	
B	28 (49.1)	83 (41.1)	0.37	25 (43.8)	
AB	4 (7)	8 (3.9)		1 (1.8)	
Preoperative bridge, no., (%)					
Ventilator	3 (5.3)	7 (3.5)		2 (3.5)	1
ECMO	0	9 (4.4)	0.27	1 (1.7)	
Intraoperative, no. (%)					
ECC	31 (54.4)	126 (62.4)	0.29	50 (87.7)	<0.001
ECMO	12 (21.1)	31 (15.4)	0.32	0	<0.001
Sternotomy	32 (56.1)	124 (62.4)	0.54	50 (87.7)	<0.001
Bilateral thoracotomy	15 (26.3)	48 (23.8)	0.73	7 (12.3)	0.09
Clamshell	10 (17.5)	30 (14.8)	0.68	0	<0.001

EVLP, *ex vivo* lung perfusion; no., number; %, precent; IQR, interquartile range; y, years; BMI, body mass index; COPD, chronic obstructive pulmonary disease; NSIP, non-specific idiopathic pneumonia; ECMO, extracorporeal membrane oxygenation.

^a^ Other diagnosis included: sarcoidosis, lymphangioleiomyomatosis, Sjögren's syndrome and non-cystic fibrotic bronchiectasis.

^b^ Prioritized listing indicates to patients with higher priority than standard on lung transplant waiting list.

## Results

### Demographics

**Figure 1:**
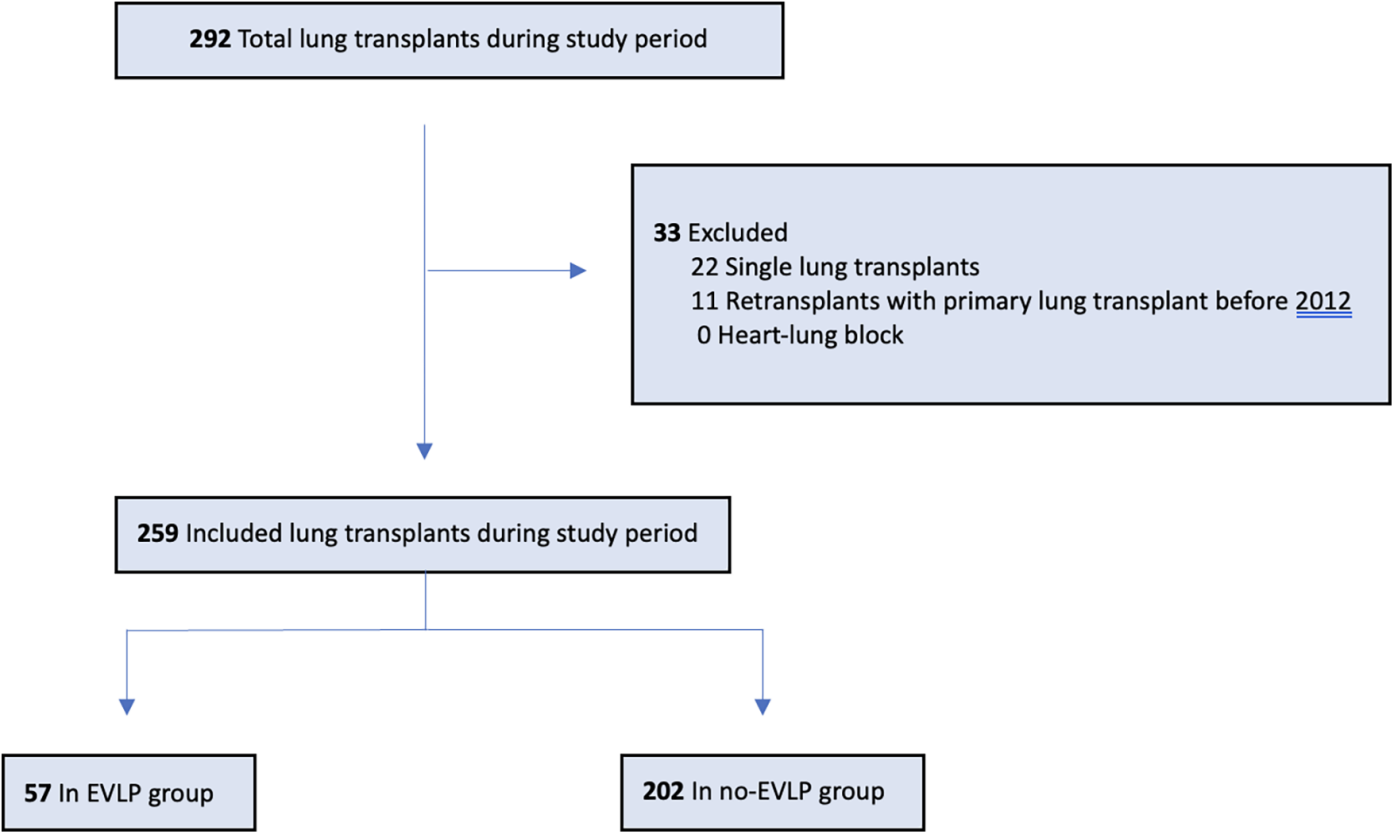
Patients included in the study. EVLP, *ex vivo* lung perfusion.

A total of 259 lung transplants were included during the study period, of which 57 were recipients of EVLP-treated donor lungs (Figure 1). The actual number of EVLP-treated donor lungs during the study period was 70. Of these, 13 were declined for not fulfilling the standard post-EVLP criteria for transplantation, thus making the EVLP utilization rate of 81.4%. The median follow-up time was 1,257 days (IQR 501–2,102) in the EVLP group, 1,462 days (IQR 674–2,264) in the unmatched non-EVLP group (*P* = 0.31), and 2,184 days (IQR 741–3,423) in the matched non-EVLP group (*P* = 0.002).

### Donor characteristics

Donor baseline characteristics are presented in Table 1. There were no statistically differences in donor sex, age, abnormalities on chest radiography, or cause of death between unmatched and matched cohorts in the EVLP and non-EVLP groups. In the unmatched cohort, the EVLP group donors had a significantly higher median BMI than the non-EVLP group donors (26.8 (IQR 24.3–29.9) vs. 24.9 (IQR 22.5–27.7), *P* < 0.05), higher proportion of donors with a history of smoking (28 of 57 (49.1%) vs. 63 of 202 (31.2%), *P* = 0.02), and lower P/F ratio (199 (IQR 143–277) vs. 366 (IQR 294–435), *P* < 0.001). The trend of BMI and P/F ratio stayed the same when the EVLP group was compared with matched non-EVLP group (BMI 26.8 kg/m^2^ (IQR 24.3–29.9) vs. 24.5 kg/m^2^ (IQR 22.4–26.9), *P* < 0.05; P/F ratio: 199 (IQR 143–277) vs. 362 (IQR 282.5–363), *P* = 0.01), whereas the proportion of donors with a history of smoking became equivalent between the groups (28 of 57 (49.1%) vs. 28 of 57 (49.1%), *P* = 1). The total median preservation time was significantly prolonged in the EVLP group compared with the unmatched non-EVLP group (661 min (IQR 575–766) vs. 390 min (IQR 300–449), *P* < 0.001) and the matched non-EVLP group (330 min (IQR 279–416), *P* < 0.001).

### Recipient characteristics

The characteristics of recipients receiving EVLP-treated and non-EVLP treated donor lungs are shown in Table 2. Baseline demographic characteristics were similar among both groups in the unmatched and matched cohorts. The most common indication for lung transplantation in the unmatched cohort of the groups was COPD/Emphysema (EVLP cohort, 19 of 57 (33.3%); non-EVLP cohort, 59 of 202 (29.2%)) followed by alpha-1-antitrypsin deficiency in the non-EVLP arm (non-EVLP arm, 32 of 202 (15.8%); EVLP arm, 3 of 57 (5.3%)) and idiopathic pulmonary fibrosis in the EVLP group (EVLP group, 10 of 57 (17.6%); non-EVLP group, 22 of 202 (10.9%)). In the matched EVLP and non-EVLP cohorts, COPD/Emphysema remained the most common indication for lung transplantation (EVLP cohort, 19 of 57 (33.3%); non-EVLP cohort, 22 (38.5%)) followed by cystic fibrosis in the non-EVLP group (non-EVLP arm, 9 of 57 (15.8%); EVLP arm, 8 of 57 (14%)) and idiopathic pulmonary fibrosis in the EVLP group (EVLP group, 10 of 57 (17.6%); non-EVLP, 5 of 57 (8.8%)). The percentages of patients bridged to lung transplant with ventilator were 5.3% (3 of 57), 3.5% (7 of 202), and 3.5% (2 of 57) in the EVLP, unmatched non-EVLP, and matched non-EVLP groups, respectively. Bridging with ECMO was only seen in the unmatched (4.4%, 9 of 202) and matched (1.7%, 1 of 57) non-EVLP groups. Intraoperative variables, such as extracorporeal circulation (ECC), ECMO, and type of thoracic access, were similar in the EVLP and unmatched non-EVLP groups; however, after matching, only bilateral thoracotomy access stayed similar between the groups.

### Primary outcomes

**Figure 2:**
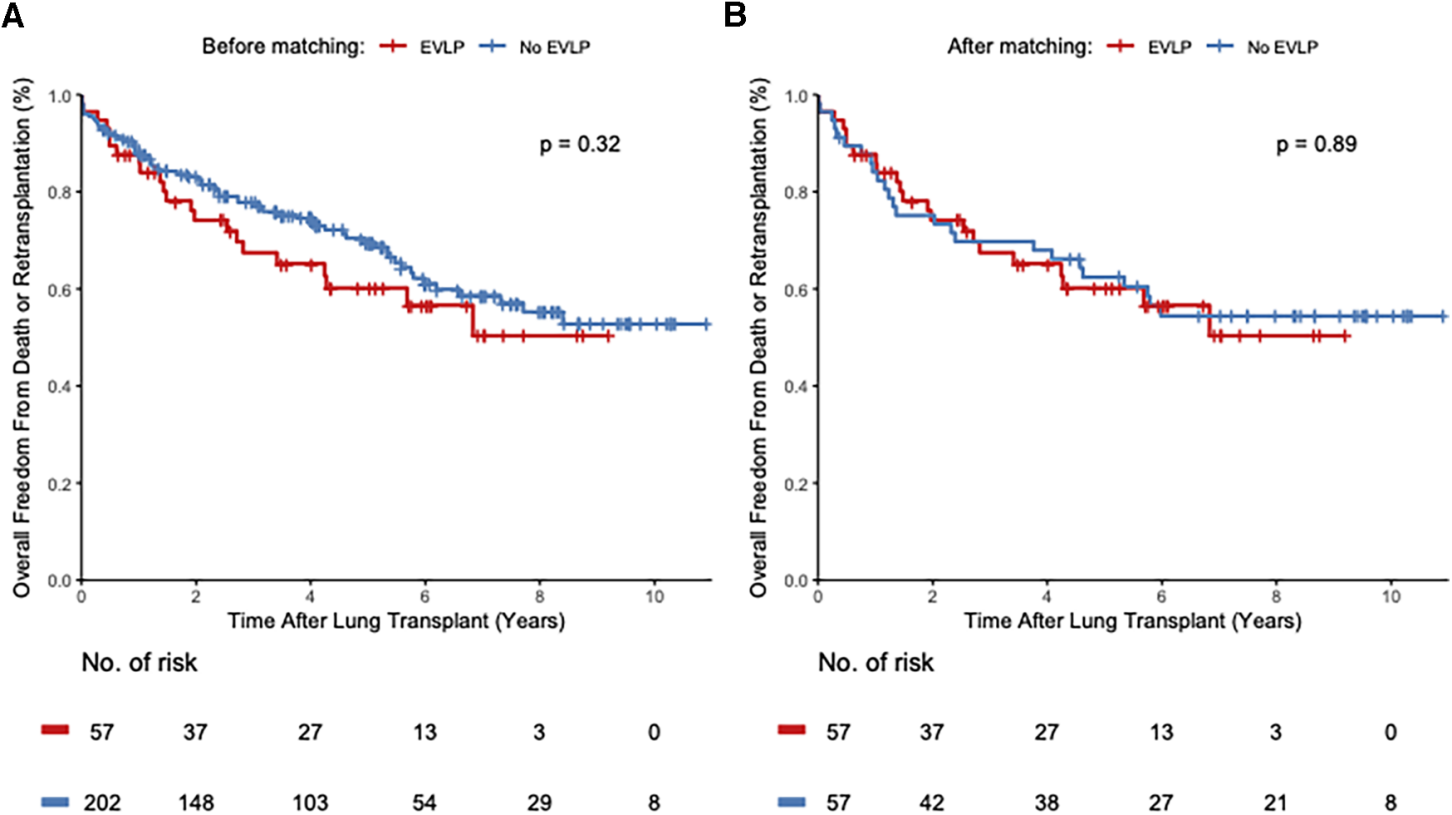
Kaplan-Meier curves depict the overall freedom from death or retransplantation of patients receiving EVLP-treated lungs compared with recipients of conventional donor lungs in the unmatched (**A**) and the propensity score matched cohort (**B**). The *y*-axis depicts the proportion of patients free from death or re-transplantation. The *x*-axis shows years after lung transplantation. The number of patients at risk is shown below the *x*-axis at every 2-year time point. %, percent; no., number; *p*, *P*-value.

Overall freedom from death and retransplantation was similar among the groups in the unmatched and matched cohorts with a HR of 1.28 (95% confidence interval (CI) 0.79–2.07, log-rank *P* = 0.32) and 1.06 (95% CI 0.59–1.89, log-rank *P* = 0.89) for the EVLP group compared with the non-EVLP group, respectively (Figures 2B). EVLP era-based overall freedom from death and retransplantation was also equivalent among these two groups in the unmatched and matched cohorts (Supplementary Materials A1–A3). Estimated freedom of death and retransplantation in the unmatched cohort was 69.7% vs. 77.3% at 3 years, 60.2% vs. 68.8% at 5 years, and 56.6% vs. 53.2% at 10 years after transplantation between the EVLP and non-EVLP groups, respectively. In the matched cohort, the estimated freedom of death and retransplantation was 69.7% vs. 68% at 3 years, 60.2% vs. 60.5% at 5 years, and 56.6% vs. 54.4% at 10 years after transplantation between the EVLP and non-EVLP groups, respectively.

**Figure 3:**
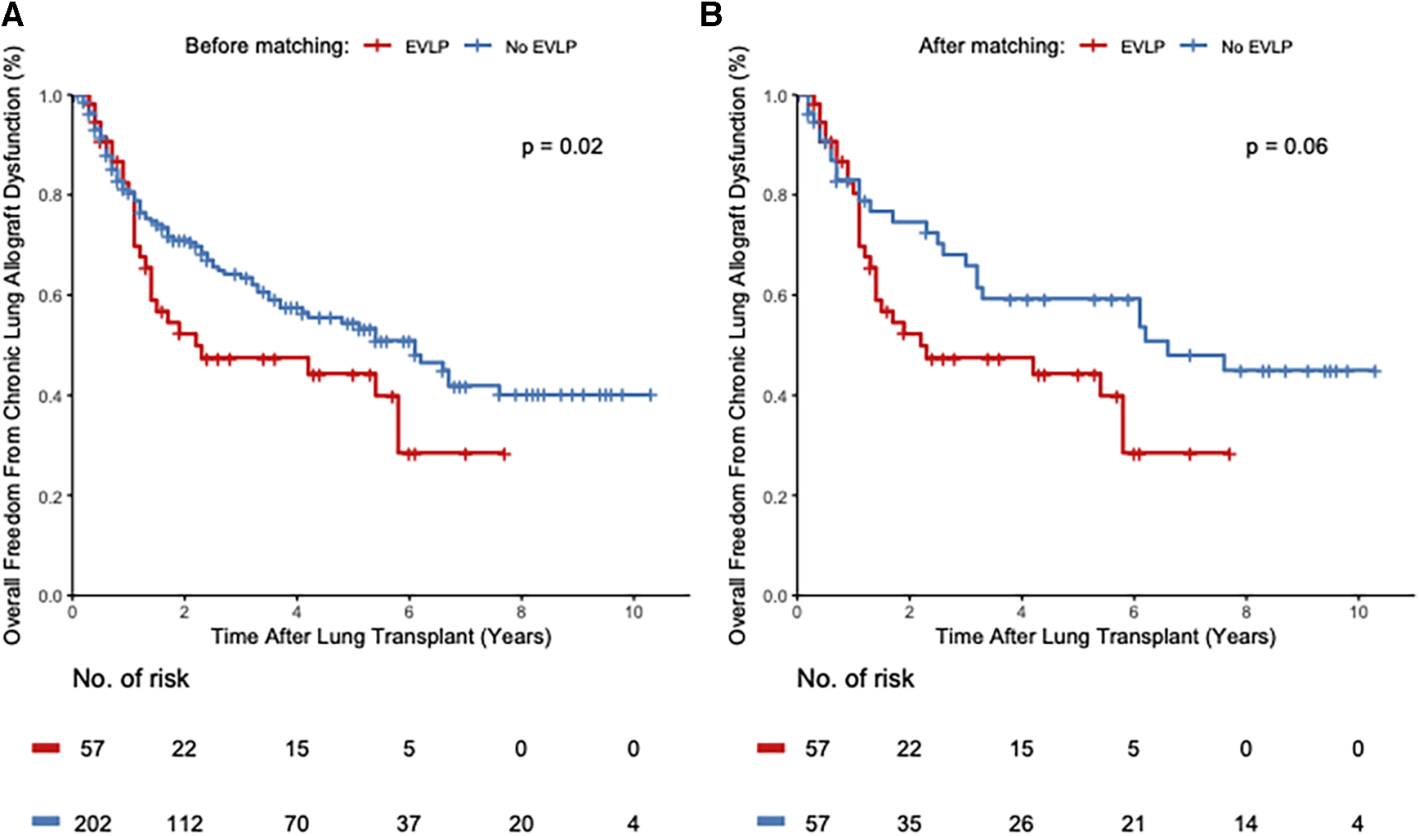
Kaplan-Meier curves depict the overall freedom from chronic lung allograft dysfunction (unadjusted for competing risk bias) of patients receiving EVLP-treated lungs compared with recipients of conventional donor lungs in the unmatched (**A**) and the propensity score matched cohort (**B**). The *y*-axis depicts the proportion of patients free from chronic lung allograft dysfunction. The *x*-axis shows years after lung transplantation. The number of patients at risk is shown below the *x*-axis at every 2-year time point. %, percent; no., number; *p*, *P*-value.

Overall freedom from CLAD before adjusting for competing bias was significantly different between the groups in the unmatched cohort, with a HR of 1.64 (95% CI 1.07–2.52, log-rank *P* = 0.02) for the EVLP group compared with the non-EVLP group (Figure 3A, Table 3). After adjusting for competing risk bias, the cumulative CLAD incidence in the unmatched cohort was similar between the groups, with a HR of 0.72 (95% CI 0.48–1.1, log-rank *P* = 0.13) for the EVLP group compared with the non-EVLP group (Table 3). In the matched cohort, overall freedom from CLAD was not different between the groups, with a HR of 1.69 (95% CI, 0.97–2.95, log-rank *P* = 0.06) before adjusting for competing bias (Figure 3B, Table 3). The cumulative CLAD incidence after adjusting for competing bias in the matched cohort was equally insignificant, with a HR of 0.91 (95% CI 0.37–2.215, log-rank *P* = 0.83) (Table 3). The incidence of EVLP era-based cumulative CLAD was significantly higher during the years 2012–2014 in the EVLP group compared with the non-EVLP group in the unmatched cohort (HR 1.42, 95% CI 1.19–1.92, log-rank *P* = 0.03); however, this difference was not seen in the matched cohort between these two groups (HR 1.49, 95% CI 1.11–3.04, log-rank *P* = 0.32) (Supplementary Material B1). No difference in cumulative CLAD was demonstrated in later years between these two groups in the unmatched and matched cohorts (Supplementary Materials B2, B3). The estimated CLAD rate in the unmatched cohort was 36% vs. 11.8% at 3 years, 42% vs. 13.5% at 5 years, and 54% vs. 15.9% at 10 years after transplantation between the EVLP and non-EVLP groups, respectively. In the matched cohort, the estimated CLAD was 36% vs. 10.1% at 3 years, 42% vs. 12% at 5 years, and 54% vs. 17% at 10 years after transplantation between the EVLP and non-EVLP groups, respectively. There was no difference in baseline median FEV_1_ between the unmatched (2.6 (IQR 2.1–3.7) vs. 2.6 (IQR 2.0–3.4), *P* = 0.52) and matched (2.6 (IQR 2.1–3.7) vs. 2.4 (IQR 1.8–3.2), *P* = 0.25) EVLP and non-EVLP groups, respectively.

**Table 3:** Hazard ratio in EVLP group compared with non-EVLP group.

	Unmatched		Matched	
	Hazard ratio (95% CI)	*P*-value	Hazard ratio (95% CI)	*P*-value
Overall freedom from CLAD (unadjusted)	1.64 (1.07–2.52)	0.02	1.69 (0.97–2.95)	0.06
Cumulative CLAD incidence (adjusted)	0.72 (0.48–1.1)	0.13	0.91 (0.37–2.21)	0.83

EVLP, *ex vivo* lung perfusion; CLAD, chronic allograft lung dysfunction; CI, confidence interval; KM, Kaplan-Meier.

### Secondary outcomes

There were no significant differences in secondary outcomes among the groups (Table 4). The incidence of postoperative outcomes such as PGD > Grade 1 at 72 h, ECMO, ventilation, tracheotomy, length of ICU stay, length of hospital stay, and biopsy-proven acute rejection episodes were similar across the groups in the unmatched and matched cohorts.

**Table 4:** Recipient secondary outcomes.

Variables	Groups				
	Unmatched			Matched	
	EVLP (*n* = 57)	Non-EVLP (*n* = 202)	*P*-value	Non-EVLP (*n* = 57)	*P-*value
Postoperative outcomes					
PGD > Grade 1 at 72 h, no. (%)	10 (17.5)	20 (9.9)	0.09	4 (7)	0.05
ECMO post LTx, no. (%)	3 (5.3)	13 (6.4)	1.00	1 (1.7)	0.06
Ventilator, median (IQR), day	1 (0–3)	0 (0–2)	0.09	0.5 (0–1)	0.12
Tracheotomy, no. (%)	8 (14.0)	23 (11.4)	0.65	5 (8.7)	0.08
ICU stay, median (IQR), day	4 (2–9)	3 (2–9)	0.22	3 (2–5)	0.29
Hospital stay, median (IQR), day	31 (23–49.5)	29 (22–41.5)	0.25	32 (22–52)	0.91
Biopsy-proven rejection episodes, median (IQR)	1 (1–1.5)	1 (1–2)	0.16	1 (1–2)	0.11
Development of CMV infection, no. (%)	21 (36.8)	81 (40.1)	0.75	25 (43.9)	0.53

EVLP, *ex vivo* lung perfusion; PGD, primary graft dysfunction; h, hours; ECMO, extracorporeal membrane oxygenation; LTx, lung transplant; no., number; %, percent; IQR, interquartile range; d, days; ICU, intensive care unit; CMV, cytomegalovirus.

## Discussion

The long-term follow-up results of the EVLP technique have been limited by its relatively short clinical availability. Consequently, the majority of the current evidence is based on short-term and medium-term outcomes of the strategy mandating long-term outcomes for its more liberal clinical application regardless of different EVLP protocols (15–23). This study delineates 10 years of EVLP outcomes, with one of the main results demonstrating no difference in overall freedom from death and retransplantation between recipients who received EVLP-treated donor lungs compared with conventional donor lung recipients in the unmatched and matched cohorts. This finding suggests that the results achieved from the EVLP evaluation are legitimate as markers for lung function, thus making the strategy a well-founded cornerstone to further increase the donor pool. Limited prior studies investigating the long-term outcomes of EVLP using the LUND, Toronto, and OCS protocols have demonstrated similar results to our study, contributing significantly to much-required additional evidence of long-term results of the strategy (13, 24–26).

The development of CLAD is another long-term outcome of major concern in lung transplant as it is associated with inevitable poor outcomes (30). In the present study, the overall freedom from CLAD over time in the unmatched cohort was significantly different between the groups with higher CLAD rates in the EVLP group compared to the non-EVLP group. However, this result was mostly attributed to the fact that our CLAD data set contained a competing risk factor—patient death before and/or unrelated to CLAD development—leading to a significant bias toward the overestimation of CLAD in both groups. After adjusting for this bias, the cumulative CLAD incidence was similar in patients with donor lungs treated with and without EVLP, thus substantiating the fact that application of the EVLP strategy may not be detrimental compared to the conventional donor lung preservation method. In fact, these findings were additionally supported by no difference in overall freedom from CLAD over time and cumulative CLAD incidence between the matched cohorts of the EVLP and non-EVLP groups. Furthermore, the findings were supplemented by similar mean baseline FEV_1_ values between the groups in the unmatched and matched cohorts, a factor that has previously been identified as a confounder in terms of time to CLAD (31). Our findings are in line with other medium-term and long-term CLAD studies, including the results of other EVLP protocols, with our results providing additional evidence of EVLP as a non-deleterious method to utilize initially rejected donor lungs (13, 15, 16, 19–26).

In recent years, the use of EVLP for extended criteria donor lungs has progressively increased in the majority of the centers (7, 13, 15, 17, 18, 24). This has also been the case in our center, leading to an increased uptake of EVLP cases. EVLP era-based sub-analyses in this study did not demonstrate any difference in overall freedom from death and retransplantation in any of the eras between the EVLP and non-EVLP groups in the unmatched and matched cohorts. This finding is reassuring in terms of further validating the ability of EVLP regardless of the extended criteria donor lungs included in the EVLP group in recent year, a finding that is in keeping with previous studies of different EVLP protocols (7, 13, 17, 24). Interestingly, the present study demonstrates a significantly higher incidence of cumulative CLAD in the EVLP group compared to the unmatched non-EVLP group in the early EVLP era of 2012–2014 despite no changes in the EVLP protocol. However, this was not seen in later unmatched cohorts of EVLP eras nor in any of the EVLP eras when cumulative CLAD in EVLP era-based groups were compared with matched non-EVLP era-based groups. The significant difference in the early EVLP era of the unmatched cohort may reflect the fact that EVLP was a new method in the early EVLP era, thus both donor‒recipient selection and the EVLP execution may have been subjected to a learning process leading to suboptimal results. In fact, a previous study from our center demonstrates a similar trend in unmatched EVLP era sub-analyses, indicating the importance of not only acquiring skills in better selecting donors and recipients for an EVLP program but also mastering the EVLP technique to maintain the required expertise (32). To that point, an increased EVLP volume is essential to sustain the validity of its program and technique, which has, in our case, led to the current trend of centralizing EVLP activity in regional hubs to ensure higher EVLP numbers along with state-of-the-art EVLP expertise.

Concerns of using extended criteria donor lungs for EVLP has prompted studies to investigate additional secondary postoperative outcomes (15–23). In the present study, no difference was demonstrated between the EVLP group and non-EVLP group among selected analyzed secondary outcomes in the unmatched and matched cohorts, thus keeping our results broadly in concordance with previous studies using the LUND, Toronto, and OCS protocols (13, 15–26). Although, one previous LUND study did report a significantly longer time of ventilator and ICU stay in the EVLP group (*n* = 11) than the non-EVLP group (*n* = 47) in their early follow-up period (12); the same center demonstrated no difference in these postoperative parameters in the 4-year follow-up period, with increased patient numbers in both groups (EVLP *n* = 27, non-EVLP *n* = 145) indicating the importance of increased EVLP volume (15).

Donor lung characteristics are of paramount importance in terms of lung transplant outcomes. In particular, the association between donor smoking history and lung transplant outcomes has been examined, with studies demonstrating the poor effect of donor smoking on early and late LTx outcomes (33–35). In our study, a significantly higher proportion of donors with a smoking history was found in the EVLP donor lung group compared with the unmatched non-EVLP donor lung group. Despite that, no difference was demonstrated in the primary and secondary outcomes between these two groups in the unmatched cohorts, suggesting that transplantation after EVLP can be performed safely using pre-EVLP donor lungs, which are significantly more vulnerable than standard non-EVLP donor lungs. Interestingly, this finding may also reflect the fact that having access to EVLP changes the willingness of a surgeon to accept more suboptimal quality lungs in order to increase donor lung availability, a hypothesis that is consistent with the experiences of other centers (13, 36, 37). Although our results are in line with a previous study with overweight of donor lungs with smoking history in EVLP group, further studies are needed to elucidate the ability of EVLP in term of its exposure to extended criteria donor lungs (24). Nevertheless, smoking history in the matched cohorts of the EVLP and non-EVLP groups was similar, with no difference in primary and secondary outcomes.

One of the main criteria for evaluating donor lungs on EVLP is a decreased P/F ratio, of which the cutoff values vary between the centers (15, 16, 20). According to our protocol, a P/F ratio of <300 mmHg is an indication of evaluating donor lungs on EVLP, while other centers have an even lower cutoff value (P/F ratio < 225 mmHg) (20, 24). In the present study, the mean P/F ratio was significantly lower in the unmatched and matched cohorts of the EVLP group than the non-EVLP group, which was anticipated (15, 20, 24). Interestingly, our mean P/F ratio for the EVLP group was much lower than in studies with a cutoff P/F ratio lower than our protocol (20, 24). This suggests that the donor lungs in our EVLP cohort were actually more vulnerable, hence demonstrating that lungs with even poorer pre-EVLP function can be safely transplanted with no difference in long-term and short-term outcomes, results that have also been demonstrated in previous studies (20, 25, 26). In addition, this information could be used to potentially re-evaluate the EVLP donor acceptance criteria and further increase the number of donor lungs accepted for EVLP, which could be relevant with lungs obtained from the “circulatory death” method as they are known to have poorer PaO_2_ values (38, 39).

The cause of a decreased P/F ratio is important to identify, which, if reversible, such as atelectasis, can be reversed leading to a sufficient increase in the P/F ratio thus permitting a direct LTx or eligibility for EVLP, avoiding initial discarding of donor lungs (40, 41). The majority of the centers use varied but “gentle” lung recruitment regimes to reduce the atelectasis regardless of donor BMI or the proportion of atelectasis (15, 16, 20). However, recent studies have demonstrated that the high BMI donor group (BMI > 25 kg/m^2^) usually has substantial atelectasis contributing to a much lower P/F ratio, where “gentle” lung recruitment does not sufficiently increase the P/F ratio (40, 41). Therefore, an “aggressive” intraoperative lung recruitment (PEEP of 25–30 mmHg for 30 s) at the donor site has been introduced, which, if unsuccessful, can lead to the use of EVLP, resulting in a sufficient increase in the P/F ratio with satisfactory outcomes, thus salvaging the majority of the initial marginal lungs (40, 41). In the present study, the EVLP group had a significantly higher BMI of more than 25 kg/m^2^ with a lower P/F ratio, despite the “gentle” lung recruitment regime used in our center. This suggests that this cohort could have perhaps benefitted from “aggressive” lung recruitment, which was not applied as this is not part of our protocol. However, it would have been interesting to determine whether “aggressive” lung recruitment would increase the P/F ratio in this cohort and how many of these donor lungs would have been eligible for a direct transplantation. To date, donor BMI has not been considered as a marginal donor criterion (42). However, targeting high-BMI donors with a low P/F ratio might be advantageous to increase direct transplantable donor lungs, with EVLP as a backup.

This study has several limitations. First, this is a retrospective single-center study, hence it is subjected to the usual caveats of interpretating data of such design. Randomized control trials in this area are difficult, as transplanting initially rejected or marginal donor lungs as a control arm would be ethically difficult to justify (36). Second, there is a known heterogeneity in the management of donor lungs before and after 2020 due to the change in protocol. This may not exclude the potential effects on transplant outcomes. Third, we did not perform *a priori* power calculations as our patient sample was limited to the transplants we have performed. Adding to this point, the small sample size in the present study is an additional limitation, especially considering the study period was 10 years. At last, the marginal donor lungs in our study were evaluated on EVLP on the assumption that they would be eligible for transplantation after EVLP, otherwise discarded. Thus, there is an inherent selection bias toward donor lungs assessed on EVLP, making them less vulnerable with potentially better outcomes than expected if all severely marginal lungs with no contradictions were treated on EVLP.

In conclusion, this 10-year follow-up study demonstrates that transplantation of marginal donor lungs after evaluation on EVLP is non-detrimental compared to conventionally preserved donor lungs in unmatched and matched cohorts in terms of mortality, retransplantation, cumulative CLAD incidence, and secondary outcomes, thus making EVLP a reasonable option to salvage marginal donor lungs for transplantation. Although the early EVLP era of 2012–2014 in the unmatched cohort had a significantly higher cumulative CLAD incidence, no such finding was demonstrated in later unmatched EVLP eras, indicating that a higher EVLP volume is essential to maintain the state-of-the-art EVLP expertise, which concomitantly ensures the validity of the EVLP program and technique. No difference was demonstrated in any of the EVLP eras in the matched cohort.

## Data Availability

The raw data supporting the conclusions of this article will be made available upon request by the authors, without undue reservation.
