# Supplementary material for: Long-term outcomes of lung transplantation with ex vivo lung perfusion technique
Source: Front Transplant. 2024 Feb 6;3:1324851. doi: 10.3389/frtra.2024.1324851 (PMC11235351; doi:10.3389/frtra.2024.1324851)
Supplement: Supplementary file 1 [file Datasheet1.docx]

**Supplementary Materiel:**

**A: Era based sub-analysis of overall freedom from death and re-transplantation for EVLP group compared to non-EVLP group:**

**A1: 2012-2014**

| Unmatched | | Matched | |
| --- | --- | --- | --- |
| HR (95% Confidence Interval) | P value | HR (95% Confidence Interval) | P value |
| 1.97 (0.96 - 4.04) | 0.06 | 1.26 (0.48 - 3.33) | 0.63 |

**A2: 2015-2018**

| Unmatched | | Matched | |
| --- | --- | --- | --- |
| HR (95% Confidence Interval) | P value | HR (95% Confidence Interval) | P value |
| 1.17 (0.55 – 2.47) | 0.67 | 0.96 (0.39 – 2.17) | 0.85 |

**A3: 2019-2021**

| Unmatched | | Matched | |
| --- | --- | --- | --- |
| HR (95% Confidence Interval) | P value | HR (95% Confidence Interval) | P value |
| 0.64 (0.14 – 2.9) | 0.57 | 0.67 (0.12 – 3.54) | 0.64 |

**B: Era based sub-analysis of overall freedom from Chronic Lung Allograft Dysfunction (CLAD) and Cumulative CLAD incidence for EVLP group compared to non-EVLP group:**

**B1: 2012-2014**

|  | Unmatched | | Matched | |
| --- | --- | --- | --- | --- |
|  | HR (95% Confidence Interval) | P value | HR (95% Confidence Interval) | P value |
| Overall freedom from CLAD | 3.25 (1.55 – 6.82) | 0.0017 | 4.18 (1.3 - 13) | 0.01 |
| Cumulative CLAD | 1.42 (1.19 – 1.92) | 0.03 | 1.49 (1.11-3.04) | 0.32 |

**B2: 2015-2018**

|  | Unmatched | | Matched | |
| --- | --- | --- | --- | --- |
|  | HR (95% Confidence Interval) | P value | HR (95% Confidence Interval) | P value |
| Overall freedom from CLAD | 1.12 (0.60-2.09) | 0.71 | 0.96 (0.45-2.03) | 0.92 |
| Cumulative CLAD | 0.92 (0.51-1.69) | 0.8 | 1.01 (0.49-2.08) | 0.97 |

**B3: 2019-2021**

|  | Unmatched | | Matched | |
| --- | --- | --- | --- | --- |
|  | HR (95% Confidence Interval) | P value | HR (95% Confidence Interval) | P value |
| Overall freedom from CLAD | 1.0 (0.37 – 2.71) | 0.98 | 0.67 (0.22-2.01) | 0.47 |
| Cumulative CLAD | 0.96 (0.39 – 2.38) | 0.94 | 1.66 (0.57-4.8) | 0.35 |
